# Inflammation in Prostatic Hyperplasia and Carcinoma—Basic Scientific Approach

**DOI:** 10.3389/fonc.2017.00077

**Published:** 2017-04-25

**Authors:** Božo Krušlin, Davor Tomas, Tihana Džombeta, Marija Milković-Periša, Monika Ulamec

**Affiliations:** ^1^Department of Pathology, School of Medicine, University of Zagreb, Zagreb, Croatia; ^2^Department of Pathology, Clinical Hospital Centre Sestre Milosrdnice, Zagreb, Croatia; ^3^Department of Pathology, University Hospital for Tumors, Zagreb, Croatia

**Keywords:** inflammation, benign prostatic hyperplasia, prostatic carcinoma, inflammatory cells, cytokines

## Abstract

Chronic inflammation is associated with both benign conditions and cancer. Likewise, inflammatory cells are quite common in benign prostatic hyperplasia (BPH) and prostatic tissue harboring cancer. Triggers that activate inflammatory pathways in the prostate remain a subject of argument and are likely to be multifactorial, some of these being bacterial antigens, different chemical irritations, and metabolic disorders. Acute and chronic inflammation in prostate leads to accumulation of immunocompetent cells, mainly T lymphocytes and macrophages, but also neutrophils, eosinophils, and mast cells, depending on the type of offending agent. Inflammatory processes activate hyperproliferative programs resulting in nodules seen in BPH, but are also important in creating suitable microenvironment for cancer growth and progression. Inflammatory cells have mostly been shown to have a protumoral effect such as tumor-associated macrophages, but some cell types such as mast cells have antitumoral effects. This review outlines the recent findings and theories supporting the role of inflammatory responses as drivers of both benign and malignant epithelial processes in the prostate gland.

## Introduction

The vast majority (90%) of all cancers are linked to somatic mutations and environmental factors. It is assumed that chronic inflammation, including infections and autoimmune diseases, as well as inflammatory conditions of uncertain origin are important in creating suitable microenvironment for cancer growth and progression. Long-lasting inflammation is also present in many benign conditions but without apparent proof of cancer growth (1–5).

Several multi-center studies demonstrated that the presence of various polymorphisms in genes coding for interleukins (ILs) such as IL-1, IL-6, and/or IL-8 is strongly associated with increased risk of cancer development. However, the role of inflammatory signals in tumor initiation is difficult to define due to multitude of molecules and mechanisms involved and also owing to lack of appropriate experimental models (1–4).

Benign prostatic hyperplasia (BPH) has been shown to be associated with inflammatory environment, and recent studies suggest there may be inflammation-related prostate cancer growth as well. In this review, we will display short reflection on such studies.

## BPH and Inflammation

**Figure 1:**
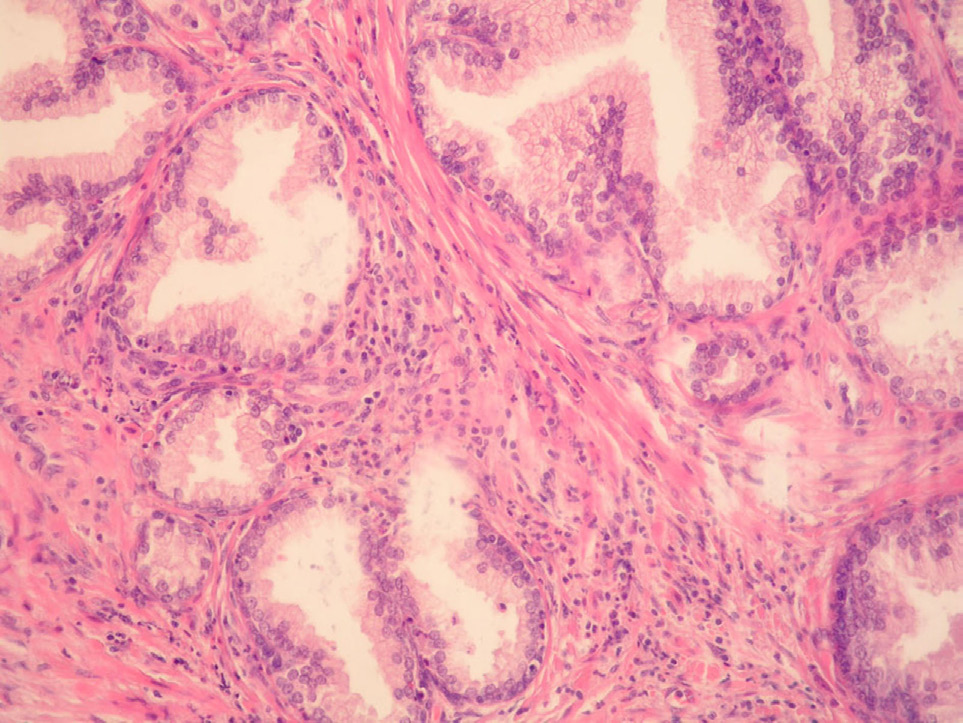
**Benign prostatic hyperplasia**. Inflammatory cells, mostly mononuclears and sparse granulocytes can be seen in the stroma surrounding benign prostatic glands. There are also a few intraepithelial granulocytes (HE, ×200).

Benign prostatic hyperplasia is a chronic, slowly progressive disease, characterized by growth of epithelial and stromal cells from the transition zone and periurethral areas. It is almost physiological process, present in most men after 50 years and some amount of chronic inflammation is inevitably present (Figure 1) (6–8). It is the most common cause of lower urinary tract symptoms in men, causing a deterioration in urinary function and increased risk of urinary tract infection, as well as increased risk of acute urinary retention (9).

Benign prostatic hyperplasia is caused by changes in hormone balance and consequently in cell growth, but molecular pathways leading to this condition are still largely unknown. Inflammatory component is believed to have an important role while presence and degree of inflammation corresponds to prostate volume and weight (6, 10). The origin of inflammation in the prostate remains a subject of argument and is likely to be multifactorial. It represents a chronic process of wound healing, which activates hyperproliferative programs resulting in BPH nodules (6, 10).

Acute and chronic inflammation leads to accumulation of immunocompetent cells in the prostate, mainly T lymphocytes and macrophages. However, many other cell types may be observed, including neutrophils, eosinophils, and mast cells, depending on the type of offending agent (11). Most of the lymphocyte population around prostate glands are CD8+ T-lymphocytes while stroma mostly contains clusters of B-lymphocytes enclosed with CD4+ T-lymphocytes (8). Not only lymphocytes but also stromal and epithelial cells have cytokine receptors on membrane surface, participating in the local immune response (11–13).

Inflammatory pathways are triggered by viral or bacterial antigens, as well as different chemical irritations and metabolic disorders. Both prostate epithelial and stromal cells and inflammatory cells produce cytokines (CCL-5, CCL-2), ILs (IL-1α, IL-1β, IL-6, IL-18), and hypoxia-inducible factor-1α (HIF-1α), creating local inflammatory microenvironment (13–15). Abundant lymphoid infiltrates with a massive increase in CD4+ T-lymphocytes, as well as macrophages and mast cells, are noted in chronic prostate inflammation (9). These cells participate in pathological changes characteristic for both BPH and prostate carcinoma. There are several studies demonstrating that ILs, which have a pro-inflammatory role, may lead to initiation and progression of BPH (11, 14–18). McDowell and associates showed how inflammatory cells can be attracted to the prostate tissue microenvironment and can selectively promote the proliferation of prostate epithelial cells (19). Further studies have confirmed IL-17 to be the initiator of BPH progression *via* activation of the nuclear-factor-kappa-B (NF-κB) pathway, which leads to secretion of other pro-inflammatory cytokines, such as IL-1, IL-6, and IL-8. Steiner et al. (20) demonstrated that healthy prostates do not express IL-17, whereas prostates with inflammation and BPH do. Wang et al. (21) also found cyclooxygenase 2 (COX-2) expressed in macrophages and epithelial prostate cells within significant inflammation. Under certain conditions, if high level of T-lymphocytes is reached, surrounding cells are killed by CD8+ cytotoxic T cells and prostatic tissue is replaced by fibromuscular nodules (9, 12). Local hypoxia and inflammation also promote fibroblast to myofibroblast transformation, which leads to extracellular changes forming suitable microenvironment for continuous inflammation (9, 13, 22). Inflammation is also continuously stimulated by androgens and changes within metabolic syndromes, but exact pathways are still mostly unknown (12, 23, 24).

## Prostate Cancer and Inflammation

**Figure 2:**
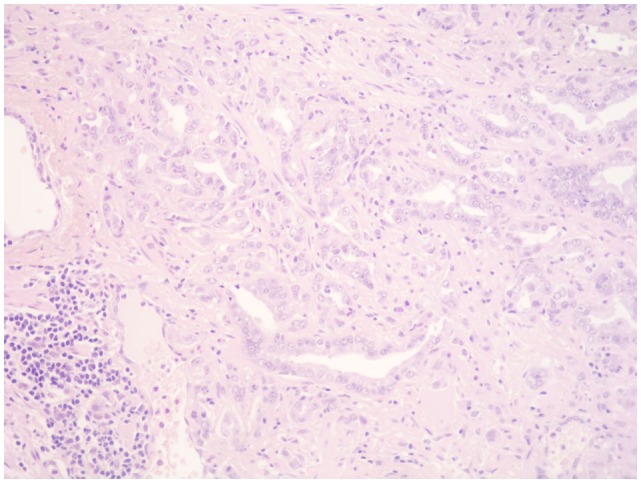
**Prostate cancer**. Scattered mononuclears can be seen between the neoplastic prostatic glands. A collection of lymphocytes, plasma cells, and macrophages can be seen at the periphery (HE, ×200).

Prostate cancer is one of well-known malignant diseases with multifactorial causes and acquired genetic and epigenetic changes (Figure 2). Chronic inflammatory microenvironment is considered to have a contribution in the development of prostate cancer. Two molecular and cellular pathways link inflammation and cancer, intrinsic end extrinsic. In the intrinsic one, oncogenes are activated and pushed to the expression of inflammation-related programs. In the extrinsic pathway, inflammation itself is the promoter of cancer development. In both scenarios, inflammatory microenvironment is present (Table 1). Transcription factors, such as TNF-α and β, Stat3, HIF-1, cytokines, and chemokines are main molecules that promote such state.

**Table 1:** **Roles of inflammatory cells and mediators in prostate cancer**.

Cell type	Chemokine	Pathway	Anti/protumoral development
Tregs, NKT	IL-2	IL-2-dependent mechanisms	– Chemokine secretion in stimulation of tumor progression
		Cell-contact dependent mechanisms	– Suppression of proliferation, cytokine release, and cytotoxic activity of NKT cells
Tumor-Tumor-associated macrophages	IFN-γ	M1 pathway	– Against tumor activity
	IL-3, IL-4, IL-10, CCL17, CCL2, CSF-1, VEGF, COX-2, PGE2	M2 pathway	– Tissue remodeling—tumor promotion toward M2 – Angiogenesis – Cancer cell proliferation
	TNF-α, IL-1, IL-6, VEGF	NF-κB	– Activation of pro-inflammatory genes (COX-2, iNOS, TNF-α, IL-1, IL-6) – Activation of antiapoptotic genes (Bcl-2, Bcl-X) – Activation of proangiogenic molecules (VEGF)
	CCM-2, MIC-1		– Tumor-promoting macrophage infiltration in prostate cancer – Promotion of local invasion and metastases
	STAT-3	Androgen receptor—CCL4-STAT3	– Downregulation of p53/PTEN tumor suppressors – Promotion of epithelial-to-mesenchymal transition pathways
	IL-1, TNF, IL-6, IL-18		– Microenvironmental niches for preserving tumor cells
	Inflammasome	Inflamm. regulators	– May be cancer specific
Neutrophils (high level)		Neutrophil–lymphocyte ratio	– Poor overall survival and recurrence-free survival
Mast cells (low intratumoral count)			– Higher risk of prostate cancer recurrence

*Tregs, regulatory T cells; NKT, natural killer T cells; IL, interleukin; IFN-γ, interferon γ; CCL, chemokines; CSF-1, macrophage colony-stimulating factor 1; VEGF, vascular endothelial growth factor; COX-2, cyclooxygenase-2; PGE2, prostaglandin E2; NF-κB, transcriptional regulation family; CCM-2, monocyte chemoattractant protein 1; MIC-1, macrophage inhibitory cytokine; STAT-3, signal transducer and activator 3*.

Different studies showed the association between specific immune cells and prostate cancer, especially CD3+, CD4+, and CD8+ cells in tissue obtained by prostatectomy or biopsy and found some of these cells to be pro-tumorigenic (25–27). For a detailed table summarizing the findings of the role of immune cells in prostate cancer, please see a review by Strasner and Karin (26). There are also studies demonstrating protective inflammatory activity in prostate cancer, showing that subsets of T-cells may play an important role in immune reaction to prostate cancer (26, 28). Similar results were obtained on animal models (29). Regulatory T cells (Tregs) and NKT cells are two subpopulations of T lymphocytes that independently regulate innate and adaptive immunity. Activated NKT cells can influence the function of Tregs through IL-2-dependent mechanisms. Tregs can also suppress proliferation, cytokine release, and cytotoxic activity of NKT cells by cell-contact-dependent mechanisms. Tumor cells stimulate T cells and interact with the tumor-infiltrative innate immune cells secreting cytokines that are crucial for T-cell differentiation (11). Mrakovčić-Šutić et al. (11) showed that, in prostate cancer, number of Tregs is elevated and percentage of NKT cells is slightly diminished compared to patients with BPH (11). Although the exact mechanism is still unknown, increased infiltration of prostate tissue with T regulatory cells seems to stimulate the tumor to secrete different chemokines that attract these cells in the prostate tissue where they may contribute to tumor progression.

It is believed that tumor-associated macrophages (TAMs) are one of the most important inflammatory cells due to their capability to promote anti- and protumor conditions. Macrophages and polymorphonuclears can polarize in response to different ILs and interferon and as such are important for tumor promotion (2, 3). Polarized cells can show anti and pro-tumoral response and in most studies inflammatory cells in tumor tissue showed protumoral polarization, which depends on pathways guided by different types of ILs.

In classical macrophage activation pathway (M1), macrophages are exposed to interferon-γ and react against tumor activity. Macrophages can also act through M2 activation pathway. In that case, they respond to IL-3 or IL-4 and promote tissue remodeling in a way of tumor promotion (2, 30). In most, but not all analyzed tumors, TAMs have an M2-like phenotype, which leads to the main question—which are the pathways leading to development of pro-tumoral inflammatory environment (31, 32)?

There are studies showing that extracellular matrix components, IL-10, chemokines CCL17 and CCL2, and CSF-1 produced by tumor cells drive macrophages toward M2 mode (2, 31, 32). Different pathways are shown to produce such changes in different tumor types (32–34).

The infiltration of macrophages and immune suppressor cells was found to have a positive correlation to prostate cancer progression (27, 35, 36). There is some evidence that TAM count is important prognostic factor for clinical outcome and recurrence of prostate cancer (26, 37). TAMs are also related to increased production of mediators that promote angiogenesis [vascular endothelial growth factor, COX-2-derived prostaglandin E2], as well as cancer cell proliferation. NF-κB is important in these cellular alterations and is associated with insensitivity to growth inhibition, resistance to apoptotic signals, angiogenesis, tissue invasion and metastasis (38–40). It activates pro-inflammatory genes (COX-2, iNOS, TNF-α, IL-1, and IL-6) in tumor and tumor-associated cells and surrounding tissue, as well as antiapoptotic (Bcl-2, Bcl-X) and proangiogenic molecules (vascular endothelial growth factor). Hypoxia appears to impact NF-κB signaling in TAMs, activating their pro-tumor orientation (41). Hypoxia and inflammation are both characteristics of prostate tumor microenvironment so HIFs and NF-κB are the key regulators of response to those stresses. Androgen and estrogen receptors also interact with this signaling pathway (42).

Monocyte chemoattractant protein 1 (CCM-2) is also a chemokine playing a role in tumor-promoting macrophage infiltration in prostate cancer, as well as its polarization into M2 phenotype (33). Macrophage inhibitory cytokine (MIC-1) or prostate-derived factor was described as molecule that can inhibit the secretion of TNF-α by activated macrophages and reduce tumor destruction, thus influencing the microenvironment in favor of prostate cancer growth. Increased MIC-1 concentration was found in high-grade prostate intraepithelial neoplasia and high-grade prostate cancer compared to normal prostate epithelial cells (43, 44). It was also shown that MIC-1 increases in serum with the progression of metastatic prostate cancer, in a similar way as prostate-specific antigen (PSA) (45, 46). These results are confirmed on animal mice models where increased MIC-1 gene expression correlated with increased level of prostate infiltrating lymphocytes. In similar models, germ line gene deletion of MIC-1/GDF15 resulted in increased local tumor growth. In late tumor development, MIC-1 overexpression showed promotion of local invasion and metastases (45, 47, 48).

Signal transducer and activator 3 (STAT-3) is also important due to its activation in malignant cells that stimulates proliferation, survival, and angiogenesis. STAT-3 activation in immune cells promotes differentiation and recruitment of TAMs, invasion, and tumor-promoting inflammation (49). It is a well-known driver of premalignant and malignant lesions in pancreatic cancer (50, 51). Prostate cell lines models, which recapitulated an interaction between immortalized prostate epithelial cells (RWPE-1 cells) and macrophages have shown that infiltrating macrophages *per se* (without additional carcinogens) can induce tumorigenesis in prostate *via* the pathway androgen receptor-inflammatory cytokine CCL4-STAT3 activation with downregulation of p53/PTEN tumor suppressors, and promotion of epithelial-to-mesenchymal transition pathways (52, 53).

The receptor for advanced glycation end products (RAGE) has also been described as an important element that drives an inflammatory milieu and some clinical studies demonstrated its strong association with the malignant potential of various cancer types (54–59). Higher RAGE expression was found in prostate cell lines compared to normal prostate epithelial cells, with various pathways of RAGE activation being proposed (60). Zhao et al. (61) investigated immunohistochemical RAGE expression, together with high-mobility group protein 1 (HMGB1) in 85 prostate cancer patient. Its high expression was related to advanced clinical stage as well as high PSA level, which suggest that the expression of RAGE and HMGB1 is associated with the progression and poor prognosis of prostate cancer (61).

Macrophages and some of their products (IL-1, TNF, IL-6, and IL-18) are also known to increase the likelihood of metastasis while creating appropriate microenvironmental niches for preserving tumor cells (62, 63).

We should also mention the emerging class of a multimeric protein group called inflammasomes, which are considered to be important regulators of inflammation. The role of inflammasomes could be cancer-specific but additional studies are needed (27).

Some other cell types, as neutrophils and mast cells were also designated as part of inflammatory response in prostate cancer (26). Tang et al. (64) performed a meta-analysis studying a predictive value of neutrophil–lymphocyte ratio (NLR) in overall survival, recurrence-free survival, and clinical features in prostate cancer patients (64). It included 9,418 patients from 18 studies. They showed that poor overall survival and recurrence-free survival were related to high pretreatment NLR. It also correlated to lymph node involvement. Localized cancer was not related to increased NLR (64).

Mast cells are present in inflammatory environment of the prostate cancer and can provide pro- and antitumoral activities (65). Hempel et al. (66) showed a protective role of intratumoral mastocytes connecting a low number of intratumoral mast cells with a higher risk of prostate cancer recurrence (66).

There are some studies that consider inflammation to be related to changes in the proliferative inflammatory atrophy as precursor of low- and high-grade prostatic intraepithelial neoplasia and cancer, but it is not likely that inflammation alone can run this pathway (67–70).

It is well known that inflammatory microenvironment is important in growth and progression of prostate tumor cells; macrophages and other immune cells show positive correlation to prostate cancer progression. It is also known that inflammation can have antitumoral effect so different inflammatory pathways and pro-inflammatory cytokines and chemokines are equally important to be included in screening of prostate cancer patients and in decision of therapy and prognosis.

Therapeutic possibilities include immunovaccines, immunomodulators, monoclonal antibodies, and adoptive T-cell therapies, meaning there are many ongoing trials targeting immune cells and their mediators in attempt to slow down prostate cancer progression. Currently investigated immunovacines include dendritic cell vaccine with a chimeric protein as a tumor-associated antigen; PSA antigen co-stimulatory molecules delivered in viral vectors; irradiated prostate cancer cell lines; adenovirus/PSA vaccine in men with recurrent cancer after local therapy and with hormone refractory cancer; DNA-based vaccines and adenoviral vector-expressing Herpes virus thymidine kinase, which targets tumor cells and is followed by anti-herpes drug (71). Immune modulators used in studies include cytotoxic T-lymphocyte-associated antigen 4 and CT-011 anti-programmed death receptor-1 for advanced stages of prostate cancer. As adoptive cell therapy, T cells genetically engineered to target cancer specific antigen NY-ESO-1 in combination with other therapy options is used (72, 73). Monoclonal antibody targeting CD20 as neoadjuvant therapy is also under investigation (26, 74).

Some of these therapies are in the phase III trial and although targeted immune therapy is promising, it needs further assessment and will probably be used in combination with other therapeutic agents and approaches.

## Conclusion

Although the pathogenesis of BPH is not fully understood, most of the recent studies strongly suggest that the T-cell activity and associated autoimmune reaction induce epithelial and stromal cell proliferation. Further understanding of the inflammatory pathways will expand the knowledge of BPH pathogenesis and potentiate screening for patients presenting with BPH-related symptoms, as well as some novel biomarkers of prostatic inflammation and treatment strategies.

Inflammatory cells are quite common in BPH and are seen in prostatic tissue harboring cancer. Recent evidence support theories that chronic inflammation and immune response might be common drivers for both diseases, at least in some cases. The exact mechanisms directing inflammatory pathways into pro-benign (BPH) or pro-malignant microenvironment are still unknown.

Nowadays, many concepts are aimed toward tumor microenvironment, such as tumor stroma molecules or inflammation/immune cells, and are trying to find suitable biomarkers in this multitude of different possible targets. Therapeutic possibilities include immunovaccines, immunomodulators, monoclonal antibodies, and adoptive T-cell therapies. Most probable treatment options will combine standard and novel therapeutic targets.

## Author Contributions

All authors participated in drafting and writing of the manuscript.

## Conflict of Interest Statement

The authors declare that the study was conducted in the absence of any commercial or financial relationships that could be construed as a potential conflict of interest.
